# Fear of Falling and Walking Quality: What Your Walking Reveals

**DOI:** 10.1093/geroni/igaa057.3374

**Published:** 2020-12-16

**Authors:** Anisha Suri, Andrea Rosso, Jessie VanSwearingen, Gelsy Torres-Oviedo, Leslie Coffman, Mark Redfern, Jennifer Brach, Ervin Sejdic

**Affiliations:** 1 Swanson School of Engineering, University of Pittsburgh, Pittsburgh, Pennsylvania, United States; 2 School of Public Health, University of Pittsburgh, Pittsburgh, Pennsylvania, United States; 3 School of Health and Rehabilitation Sciences, University of Pittsburgh, Pittsburgh, Pennsylvania, United States

## Abstract

Fear of Falling (FOF) is common among community-dwelling older adults and is associated with increased fall-risk. In this cross-sectional study we examined the relationships between FOF and factors associated with fall-risk such as gait quality, cognition, and walking-confidence. Using baseline data from older adult participants in a randomized exercise trial (N=232; age 77±6; 65% females; 40% reported FOF), we quantified the following outcome measure for (1) gait quality: harmonic ratio (smoothness) and time-frequency spatiotemporal variables from triaxial accelerometry during 6 minute walk; gait speed, step-time CoV (variability) and walk-ratio (step-length/cadence) on an instrumented walkway; (2) cognition: Trails A and B (3) walking-confidence: Gait efficacy Scale. Mann Whitney U-tests indicated individuals without FOF had better gait quality (p<0.05): greater smoothness (2.38±.58 vs 1.14±.73), speed (1.10±.15 vs 1.04±.17 m/s) and walk-ratio (.56±.07 vs .53±.08 cm/steps/min), lower step-time CoV (3.72±1.24 vs 4.17±1.66), and greater walking-confidence (89±11 vs 79±13). A random forest classifier predicted FOF with 64% (gait only) and 70% (additional variables: cognition, walking-confidence) accuracy; Gini-index based ranking indicated gait quality (smoothness vertical (V) direction, walking speed) were consistently important variables. Linear Support Vector Machine learning yielded accuracies of 60% (only gait) and 68% (with additional measures): smoothness V, mediolateral frequency bandwidth, gait speed among top 4 ranked variables in both models, and walking-confidence in the additional measures model; smoothness-V the highest weighted coefficient (-0.52). Based on these findings, interventions targeted for gait quality and walking-confidence may be important to overcome FOF and reduce fall risks.

